# Supplementary material for: Towards a Postgraduate Oncology Training Model for Family Medicine: Mixed Methods Evaluation of a Breast Oncology Rotation
Source: Curr Oncol. 2022 Sep 10;29(9):6485–95. doi: 10.3390/curroncol29090510 (PMC9497635; doi:10.3390/curroncol29090510)
Supplement: Supplementary file 1 [file curroncol-29-00510-s001.zip › curroncol-1846509-supplementary.pdf]

Towards a Postgraduate Oncology Training Model for Family Medicine: Mixed Methods Evaluation of a Breast Oncology Rotation

Supplementary Figure S1: Data Collection Time points for each Resident Group

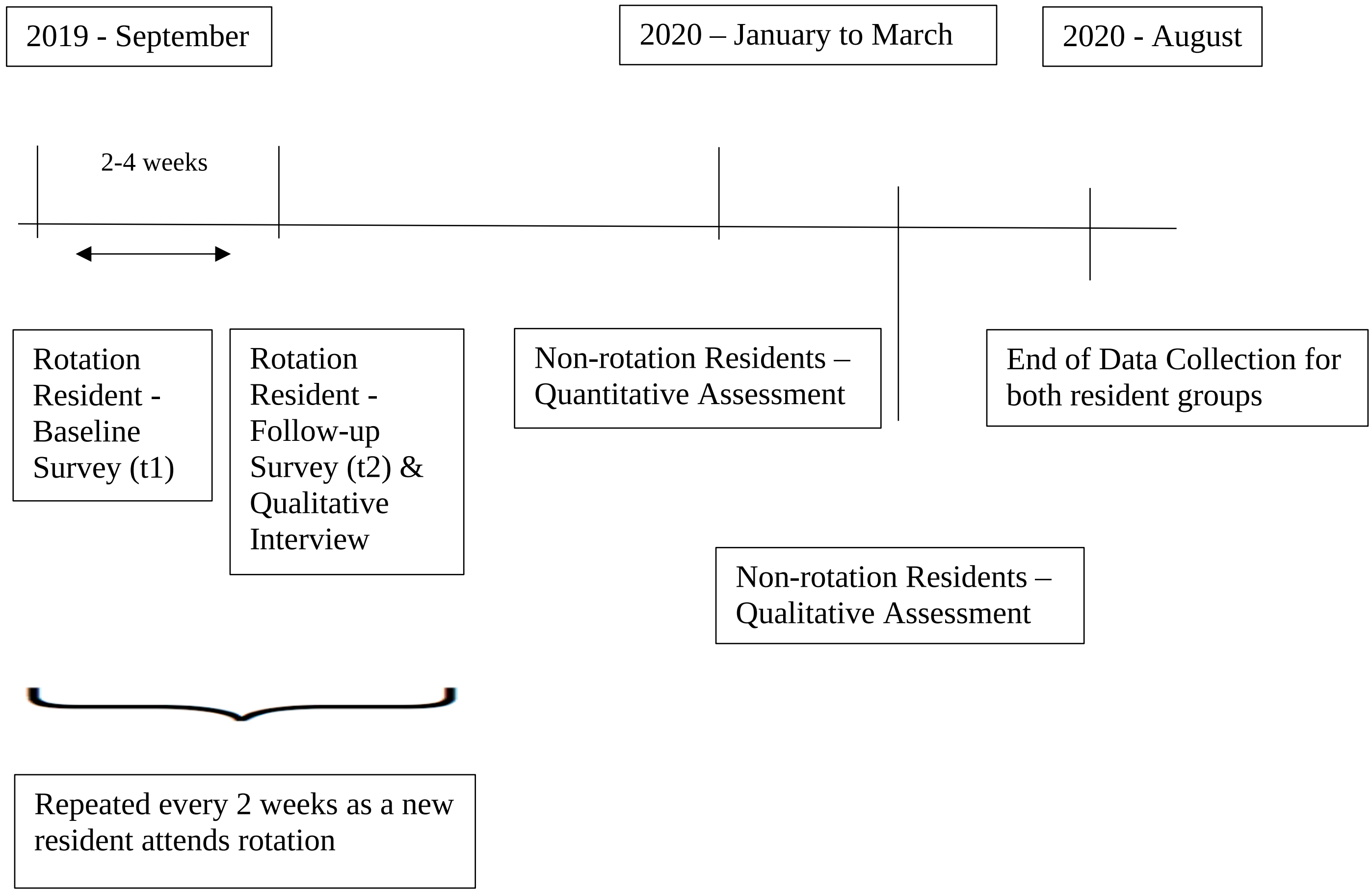

Supplementary Table S1: Breast Selective Template Schedule

Monday	Tuesday	Wednesday	Thursday	Friday
Medical Oncology (Genetics)*	Breast diagnostics (GPO)	FM - Academic half day	Survivorship ACTT clinic	Breast diagnostics (GPO)
Medical Oncology	Surgical Oncology	FM Clinical Day Back	Survivorship ACTT clinic	Surgical Oncology (rapid diagnostic)

ACTT – After Cancer Treatment Transition clinic
GPO – general practitioner in oncology
FM – Family Medicine (requirements from resident's home program)

*Residents work with a medical oncologist to counsel patients with genetic mutations about risk reduction options. If available, they shadow a genetic counsellor to learn about risk assessments & risk assessment tools (IBIS, BOADICEA, etc.)

Supplementary Table S2: Quantitative Results

**Question**	**Correct Answers (%)**		
	**Non-Rotation Residents (n=23)**	**Rotation Baseline (n=6)**	**Rotation Follow-up (n=5)**
1. Which of the following is **not** a risk factor for breast cancer?	78.3%	100%	100%
2. Which of the following statements is **correct** regarding routine population-based breast screening?	78.3%	83.3%	80.0%
**3.** All of the following criteria would qualify for high risk screening with mammogram and MRI through OBSP **except**:	34.8%	33.3%	60.0%
4. All of the following patient or family characteristics should prompt a referral for risk assessment by a genetic clinic **except:**	78.3%	83.3%	100%
5. All of the following can be used to decrease a woman's risk of breast cancer, **except:**	30.4%	83.3%	100%
6. Which of the following benign breast conditions **does NOT** increase your risk of breast cancer?	78.9%	83.3%	60.0%
7. A 33-year-old woman presents to your family medicine clinic with complaints of a new left breast mass. She has no family history of breast cancer. On examination you palpate a discrete 1 cm mobile lesion that is non-tender. The most appropriate **next step** is:	79.0%	100.0%	100%
8. A patient presents to your family medicine clinic with a 5 cm palpable mass and a large matted axillary node. Pathology from both reveals invasive ductal carcinoma, markers are pending. The most appropriate **next** step in treatment is:	89.5%	66.7%	60.0%
9. The most appropriate treatment course for a newly diagnosed 1.5 cm hormone positive, HER-2 negative, clinically node negative breast cancer includes:	78.9%	83.3%	80.0%
10. Which of the following is **false** regarding breast cancer surgical approaches?	36.8%	50.0%	80.0%
11. A woman is receiving adjuvant chemotherapy for her Stage 2 breast cancer. She comes to your office 6 days after cycle #2 feeling generally unwell with a fever of 38.7. What do you recommend?	73.7%	100.0%	80.0%
12. Which of the following statements about the side effects of breast cancer hormonal therapy is **false**?	63.2%	50.0%	100%

13. Which of the following statements about hormone therapy for the treatment of early breast cancer is **true?**	26.3%	0.0%	60.0%
14. Adjuvant chemotherapy for early breast cancer can lead to the following long-term side effects **except**:	15.8%	66.7%	60.0%
15. Current recommendations for surveillance post breast cancer diagnosis include:	31.6%	16.7%	80.0%
16. A 65-year old woman was diagnosed 10 years ago with a 2 cm node-negative breast cancer, ER/PR positive, HER2 negative. She had lumpectomy, SLNB, chemotherapy, and 5 years of letrozole (aromatase inhibitor). She comes to see you in clinic for a 2-month history of constant, slowly worsening lower back pain. She does not recall any trauma. What is your next step?	52.6%	33.3%	20.0%
17. Which of the following is **false** regarding relapse?	26.3%	0.0%	20.0%

Towards a Postgraduate Oncology Training Model for Family Medicine: Mixed Methods Evaluation of a Breast Oncology Rotation

Supplementary Table S3: Supportive Resident Quotes for Theme of Rotation Outcomes

**Theme: Rotation Outcomes**		
Category: Knowledge	Codes:<br>1.Foundation/ Framework<br>2. Referral Process and (Care) Trajectory<br>3. Roles<br>4. Management<br>5. Surveillance/ Survivorship	Rotation Residents:<br>004: Having this experience gives me more clarity about their [the patient] experience and the multiple different specialists they have to see…what the surgery was like for them … what that chemotherapy regimen looked like... later on if they're on any hormonal therapy, having a better understanding of how to counsel them around it, talk about potential side effects, and how to manage them long-term when they're on it for years.<br><br>009: Breast cancer is really not something that I've been exposed to beyond like screening. That's about it. … I wanted to get more understanding of where and what I was referring to.<br><br>002: Often the radiologist would say based on the fact that this is a cellular fibroadenoma, refer to surgery. And I think if I had not done this rotation, I would be like, 'Oh, fibroadenoma, that's perfectly fine.' And it was, but it would not have triggered an internal dialogue, to be like, 'Maybe this is a phyllodes tumour.'<br><br>Non-Rotation Residents:<br>103: I would ideally like to be comfortable with being able to at least give [patients] an overview of what to expect. For example, you're going to be referred to so-and-so and this is what they're more likely going to do…. so that I can support them both medically and emotionally.<br><br>101: There's going to be a lot of medical terms thrown around to the patient and if they're not in a healthcare field, they're not going to understand what some of those [are], like HER2 receptor…or the names of certain medications… the family doctor's role is just making sure they understand the treatments they're going through and kind of just be the translator of the medical language.<br><br>103: One area that there may be a gap… is managing the patient, for example, who may be undergoing radiation or may be undergoing chemotherapy and is presenting to your family medicine office with symptoms in between.<br><br>101: Post treatment screening people that I might have a bit more of a challenge in doing …myself, I wouldn't know the guidelines off the top of my head.
Category: Skills	Codes:<br>1.Risk Assessment<br>2. Information Synthesis/ Application<br>3. Physical Exam<br>4.Empathy	Rotation Residents:<br>002: A lot of my takeaway was learning about how risk gets stratified and the tools that oncologists and surgeons have at their disposal. The IBIS calculator actually is really cool, and you can plot where people are in real time according to their risk.<br><br>004: Now when I see reports, or even one of the imaging reports, I'm able to better process it and not feel I guess scared or nervous [about the fact that] I don't fully understand any of what happened.<br><br>004: Getting to do a bunch of breast exams was definitely great… Learning about how to feel the axillary lymph nodes properly was really helpful, and actually to feel lots of breast masses was really helpful as well.<br><br>004: In a way, I think being more empathetic to what they're going through … Especially when they talk to me about when they were going through surgery, I feel I'm better able to relate. But again, I don't feel we're ever able to fully put ourselves in the patient's shoes, but this is a way that I can get close to doing that.

Category: Comfort (Counseling)	Codes:<br>1.Counseling<br>2. Collaboration<br>3. General Comfort	Rotation Residents:<br>009: [Patients] often ask me for advice on what to do, and I just felt like I could speak from a more knowledgeable place about a very common condition and what generally happens to women who are diagnosed with breast cancer.<br><br>005: My confidence level is higher after completing the rotation. But again, if I have anything I'm not certain about I will refer to the specialist, and then I will encourage them to discuss it with a specialist.<br><br>004: Even now that I've done the rotation, I've gone back and I've seen patients who have come in with a lesion on their mammogram and then subsequently, on ultrasound. I just feel I'm more comfortable knowing how to continue to follow them and when to refer them.<br><br>003: I'm more comfortable with my breast exam for sure, more comfortable with referrals for high risk screening, how to work-up an initial breast concern, and knowing when to refer.<br>Non-rotation residents:<br>101: I'm pretty comfortable communicating a cancer diagnosis to a patient now that I've done it few times. I'm also comfortable with linking patients with resources.<br><br>103: When it comes to managing patients after they've maybe entered that system and there's more nuanced aspects to their care, that's something that I don't feel as comfortable with [or] managing patients that have ongoing treatment, whether it's radiation or chemotherapy, because I'm not entirely sure of what special considerations to keep in mind.
Category: Transferability		Rotation Residents:<br>003: I still have an appreciation for the principles of therapy, which could be surgery, chemo, or rads, but I don't know really how much I could apply that much more in detail for other cancers.<br><br>003: being comfortable, in general, with seeing people who have metastatic disease who are maybe not pursing such an aggressive approach to treatment anymore … just being in the room with them, becoming comfortable just talking about their symptoms and that kind of thing, that for sure will translate.<br><br>004: Definitely the skills are transferable and would apply to other diagnoses as well. I think the thing that was unique to breast cancer is in that process, compared to some of the other cancers, there might be a better prognosis, and the treatments may differ as well. So, I may not have as good of a grasp on other types of malignancies, for example, and therefore, may not feel as confident necessarily in terms of counselling patients. But in terms of a lot of the communication skills and reassurance counselling, I still think some of that would be transferable.<br><br>009: Certainly I felt a lot more comfortable as sad as it sounds with delivering bad news and I'm sure that translates to almost all cancers.

Supplementary Table S4: Supportive Resident Quotes for Theme of Resources and Recommendations

**Theme: Resources and Recommendations**		
Category:<br>Primer Resource		Rotation Residents:<br>004: What I think I would have really liked to have seen more of was more background information, if possible, about breast cancer screening… I would have loved to have had a little package where it talks about common chemotherapy options for patients, differences between lumpectomy and mastectomy, and some of the evidence around that, which I learned throughout my rotation. But I think if I had walked in with that information in hand, I could have started the first day right away just not feeling lost.<br><br>Non-Rotation Residents:<br>103: Certain things can be delivered didactically. A learning module, for example, working through case examples and answering questions …, I think that's a good way for me personally to learn and relate things… having an opportunity to ask questions and speak to the person, for example, a presenter or someone who's comfortable managing these things would be good. … through our rotations where you're actually, having the opportunity to manage and address these issues is probably the best hands-on way to learn how to manage these things.
Category:<br>Scheduling and Education	Codes:<br>1.Applicability to Resident<br>2.Tailored to Resident	Rotation residents:<br>002: The patients [seen in follow-up] were too stable. There wasn't anything to be done. It was either, do I want to see them again for the next year or do I never want to see them again? But I'm also someone who likes acuity. Whereas in oncology clinic, they have active breast cancer. You have to think about what staging tasks. It seems more relevant in that moment, so it's more interesting to me.<br><br>005: Maybe if we add a genetics clinic it would be relevant as well.<br><br>002: Having done the program, if you had given me a list, I probably would have replaced some of the half-days with the urgent care clinic or, sure, radiology, why not, reviewing MRIs and mammograms.<br><br>003: I am envisioning that I'll have a patient population down the road with lots of young women and eventually older women as well, so I wanted to get better at breast health.
